# Editorial: Women in digital health 2021

**DOI:** 10.3389/fdgth.2023.1261285

**Published:** 2023-08-03

**Authors:** Kathleen Gray

**Affiliations:** Centre for Digital Transformation of Health, University of Melbourne, Melbourne, VIC, Australia

**Keywords:** informatics, health, women, workforce, research

**Editorial on the Research Topic** Women in Digital Health 2021

The inaugural Frontiers in Digital Health Research Topic *Women in Digital Health 2021* was created to serve as a platform for highlighting the contribution of women scientists to this interdisciplinary and impactful field. To be considered for this collection, the first or last author had to be a researcher who identified as a woman.

Such highlights are needed to address the fact that at present less than 30% of researchers worldwide are women. Long-standing biases and gender stereotypes are known to discourage girls and women away from science-related fields, in particular STEM. However, equality in science, whether gender-related or other factors, is essential to drive sustainable development and widespread permeation. In order for conventional mindsets and systems to change and allow for more females to pursue STEM, equal opportunities must take the place of biases and stereotypes.

In Health Informatics and Digital Health specifically, worldwide recognition of women as practitioners has made strides in the peer-reviewed literature in recent times (1–3). On the other hand, evidence that describes the status of women as researchers is available only in narrower settings, for example Hartzler et al. (4).

Improving the status of women practitioners and researchers is imperative to offer a counterweight to what has been called a “heteronormative white male gaze” in health informatics and digital health (5). While women form the majority of the overall health workforce, they are rarely the major influencers of healthcare planning, including planning for digital transformation (6). Further, despite the preponderance of women among community healthcare decision makers, service users, and health information consumers, digital health innovations intended to meet the needs of women are rarely designed from a gender equity perspective and are sometimes trivialised as “femtech” (7, 8).

The articles in this Research Topic exemplify creative inquiry into compelling problems within Health Informatics and Digital Health, and illustrate diverse approaches to theory, methodology and application.

In *Which one? A suggested approach for evaluating digital health maturity models*, Woods et al. in Australia have used a systematic, consultative and iterative process to develop an assessment framework and facilitate recommendations for healthcare provider organisations to select digital maturity models.

In *Digital Biomarkers in Psychiatric Research: Data Protection Qualifications in a Complex Ecosystem*, Parziale and Mascalzoni from Italy have summarised the relevant principles of the General Data Protection Regulation, identified the main psychiatric research stakeholders, and clarified their respective data protection duties and responsibilities.

In *Building Virtual Health Training Tools for Residents: A Design Thinking Approach*, the research team Lawrence et al. from the United States applied an “empathize, define, ideate, prototype, and test” model and a mixed methods approach to co-design training tools to support residents and precepting attending physicians in virtual ambulatory care practice.

In *Young People's Use of Digital Tools to Support Their Mental Health During Covid-19 Restrictions*, researchers from Ireland Pretorius and Coyle used ads on social media to recruit people aged 18–25 to an anonymous online survey to understand the digital self-help strategies and resources they adopted during the first lockdown period.

The editors of this Research Topic—Meanne Chan of Lingnan University in Hong Kong, Annie Lau of Macquarie University in Australia, Susanna Spinsante of Marche Polytechnic University in Italy, and myself—hope that these articles will serve as a springboard for more women in research groups globally to design and disseminate science that represents the Health Informatics and Digital Health priorities and perspectives of women.
